# Supplementary material for: Are carbon emissions trading and green financial instruments synergistic? -Comprehensive quantitative research based on content analysis
Source: PLoS One. 2024 Mar 7;19(3):e0298601. doi: 10.1371/journal.pone.0298601 (PMC10919601; doi:10.1371/journal.pone.0298601)
Supplement: S1 Data — (ZIP) [file pone.0298601.s001.zip › Data/地级市Z_F工作报告.zip!/╕ú╜¿/╒─╓▌╩╨/└┤╫╘╣1⁄2╓┌║┼í╛┬φ┐╦╩2╛▌═°í┐_2019.docx]

各位代表：

现在，我代表漳州市人民政府，向大会报告政府工作，请予审议，并请市政协委员提出意见。

一、2018年工作回顾

2018年是全面贯彻落实党的十九大精神的开局之年，是改革开放40周年。以习近平同志为核心的党中央总揽“四个伟大”，领航新时代新征程，带领全党和全国各族人民朝着全面建成小康社会的宏伟目标砥砺前行，推动改革开放和现代化建设取得新的重大成就。在以习近平同志为核心的党中央坚强领导下，在省委、省政府和市委正确领导下，我们认真学习贯彻习近平新时代中国特色社会主义思想和党的十九大精神，坚决落实习近平总书记重要指示批示精神，不断强化维护核心的政治自觉和干事创业的行动自觉，按照省委十届六次七次全会、市委十一届七次八次全会的部署和省委于伟国书记对漳州提出的“当好新福建建设先锋”的指示要求，落实“六稳”工作要求，坚持以供给侧结构性改革为主线，坚持高质量发展落实赶超，实施乡村振兴战略，开展“三抓三比、十项竞赛”，坚决打好三大攻坚战，富美新漳州建设取得新成效。

2018年，全市完成地区生产总值3947.63亿元，增长8.7%；一般公共预算总收入352.06亿元，增长8.1%，地方一般公共预算收入218.75亿元，增长7.2%；规模工业增加值1531.6亿元，增长9.2%；固定资产投资2604.39亿元，增长11.4%；外贸出口533.85亿元，增长4.2%；实际利用外资45.7亿元；社会消费品零售总额1111.6亿元，增长13.1%；居民消费价格总水平上涨1.7%；城镇居民人均可支配收入35997元，增长7.9%；农村居民人均可支配收入18186元，增长9.1%；城镇登记失业率2.5%；人口自然增长率8.5‰。年度节能减排降碳任务全面完成。主要工作和成效是：

（一）聚力高质量赶超，经济增长稳中向好。凝聚全市上下的共识和力量，全力加快高质量发展落实赶超步伐，经济运行平稳健康，GDP增幅保持全省前列。坚持每季度分主题、分区域开展现场推进会，深化“五个一批”项目攻坚，实施“十百千支撑工程”和民间投资“百大千亿工程”，推动各地抓项目、扩投资、促赶超。落实“五个一批”项目2400个，新开工亿元以上项目418个、建成或部分建成150个，签约“三维”项目382个、总投资1321.48亿元。把加大工业投资作为主抓手，2018年工业投资完成880.51亿元，增长10.4%，占全市固定资产投资33.8%，金龙汽车、三宝耐腐蚀热轧卷板、福欣节镍不锈钢等项目落地开工，云霄抽水蓄能电站项目正式启动。全力以赴抓好腾龙翔鹭整改修复，PTA稳定生产，PX即将恢复生产，古雷炼化一体化一期等重大项目加快建设；古雷石化基地安全检查站建成投用，成为国内首个封关运行的石化专业园区。持续加大基础设施投入，厦漳同城大道、沿海大通道、厦蓉高速改扩建工程、九龙岭隧道等建成通车。

（二）发力供给侧改革，产业发展提质增效。坚持一业一策抓产业培育，出台促进民营企业发展、扶持做大做强服务业、旅游业、食品产业、钟表产业等系列政策，设立食品产业发展基金，用好“五项基金”，持续推动产业结构优化、质量提升。及时解决企业生产要素难题，补办企业不动产权证209本，减税降费72亿元。新增（新上）规模工业企业110家、服务业企业22家，新增科技小巨人33家、高新技术企业40家、境外上市企业2家，技改投资增长25%，第三产业比重同比提高0.8个百分点。获得“中国钟表名城”殊荣。更加有效引才聚才，人才政策红利进一步释放，新增省市引进高层次人才175人，比增316.7%。实施全域旅游三年行动计划，接待游客人数、旅游总收入分别增长20.9%和33.7%。建筑业完成产值617.62亿元，再创新高，3家建筑企业晋升总承包一级资质。全面实施乡村振兴战略，深化“345”示范工程，推进农业“两区”建设，全国畜禽养殖废弃物资源化利用现场会在漳州召开，农业绿色发展做法在全国推广；九大优势特色产业有八个全产业链产值超百亿元，新增设施农业2.4万亩，农村承包地确权登记颁证工作基本完成。

（三）打好三大攻坚战，发展短板加快补齐。把打赢脱贫攻坚战作为重大政治责任，落实“六个精准”和“两不愁、三保障”要求，实施“养鸡生蛋”工程，实现剩余2146名建档立卡贫困人口、17个贫困村全部脱贫摘帽，完成当年新增28户贫困无房户、665户危房户房屋修建，3个重点贫困县农村低保标准提高到每人每年6960元；“三千”工程资助对象覆盖全市所有贫困生，在全省率先实现科技特派员行政村全覆盖；诏安被评为“2018年度中国十佳脱贫攻坚与精准扶贫示范县市”。打好污染防治攻坚战，深化“三铁治污、提升三质”，中央和省环保、土地、海洋等督察反馈问题及时有效整改，河湖长制全面落实，“三质”水平持续提升；完成植树造林15.73万亩。防范化解金融风险攻坚战取得明显效果，全市不良贷款率2.06%，比2018年初下降1.13个百分点，政府债务余额严控在核定限额内，金融风险总体可控。

（四）深化“生态+”模式，城市颜值持续提升。坚持以“生态+”引领城市美丽转型，又好又快推进“五湖四海”项目，南山文化生态园、西院湖生态园、侨村“城市会客厅”等建成开放，西湖生态园基础设施配套全面开工，“生态+”成为百姓点赞、各界认可的漳州新名片。开展规划区范围1986平方公里“一张图”编制，取得阶段性成果。城市功能配套日趋完善，新增停车泊位4550个，中心城区第二水源、第二气源开工建设，中部生活垃圾焚烧发电厂建成投用。入选全国城市黑臭水体治理示范城市。着力开展农村人居环境整治、铁路沿线环境整治、“两违”综合治理百日会战，新增美丽乡村121个，新建乡镇污水处理设施23个，新建改造城乡公厕246座，拆违面积938.4万平方米。新（改）建农村公路260公里，200人以上自然村（贫困村）全部通硬化路。

（五）构建开放型经济，营商环境更加优化。以纪念改革开放40周年为契机，巩固开放型经济新体制综合试点试验工作成果，稳步做好机构改革，新组建的市直部门在2018年底全部挂牌成立。“生态+”等6项经验做法入选改革开放40周年“福建影响力”案例。实施提升营商环境行动计划，启动“漳州通”APP建设，企业开办时限压缩到5天以内，全市92.7%的审批服务事项实现“一趟不用跑”和“最多跑一趟”，率先在全国实施审批服务“智能秒批”新模式。加快落实证照分离、“多证合一”等商事制度改革，跨境贸易便利化水平显著提升。主动融入闽西南协同发展区建设，19个重大协作项目有序推进。对外开放持续扩大，出台促进漳台交流合作58条措施，新批台资项目82个，合同利用台资6.8亿美元；成功举办两岸企业家峰会古雷石化产业合作发展论坛、第五届海峡（漳州）茶会、第十届农博会·第二十届花博会；组团参加进博会、厦洽会、数字峰会等活动，举办首届中国食品名城（漳州）食品交易会，开展5场“漳州味·世界行”活动，积极推进与港澳经贸合作。有效应对中美贸易摩擦影响，外贸市场保持平稳。

（六）倾力普惠性民生，社会事业全面进步。积极回应代表、委员对民生事业的关切，持续加大“四难一差”补短板力度，25项为民办实事项目全部完成。坚持教育优先发展，教学质量持续提升，新建公办幼儿园83所，开工建设市职教园区、漳州一中新高中部，新增幼儿园学位22410个、中小学学位30521个。加大医疗卫生事业投入，公立医院和基层医疗机构综合改革效果显现，位于高新区的市医院新总院开工建设，新增医疗床位1200张、养老床位3426张、卫技人员2235名，实现公办村卫生所标准化建设全覆盖。成为全国第三批居家和社区养老服务改革试点市。新建保障性安居工程10084套、“安得广”惠民安居工程3378套。文化体育事业蓬勃发展，文艺精品创作再获佳绩，芗剧现代戏《谷文昌》代表福建晋京展演；《漳州海洋与渔业文化丛书》获得国家海洋优秀科技图书奖；参加第十六届省运会和第十届老健会取得好成绩。深入开展扫黑除恶专项斗争和维稳“六大专项行动”，扎实抓好中央扫黑除恶专项斗争督导组反馈问题整改，社会保持安定稳定。严格落实安全生产责任，扎实推进重点行业领域专项整治，安全生产形势平稳向好，事故总量明显下降。认真组织第四次全国经济普查，顺利完成第三次全国农业普查。妇女儿童、青少年、残疾人、外事侨务、民族宗教、档案、社会科学、地方志、防汛防台、防震减灾、红十字会、援藏援疆援宁等工作取得新进步。

（七）坚持新思想引领，自身建设有效加强。牢固树立“四个意识”，坚定“四个自信”，坚决做到“两个维护”，全面兴起“大学习”热潮，推动习近平新时代中国特色社会主义思想在漳州落地生根。坚持党的全面领导，认真落实省委“八个坚定不移”要求，坚决抓好习近平总书记重要指示批示精神贯彻落实情况“回头看”、中央和省巡视反馈问题整改。严格执行中央八项规定及其实施细则和省市实施办法，坚决反对“四风”，大力弘扬“马上就办、真抓实干”，加快建设法治政府、廉洁政府。市本级“三公”经费支出下降10.5%。顺利完成村级组织换届选举。认真执行人大决定决议，自觉接受人大、政协和社会监督，办理人大代表建议152件、政协委员提案322件，办结率100%。各民主党派参政议政、民主监督富有成效。政务“五公开”持续推进，行政监督、审计监督继续加强，党风廉政建设和反腐败工作有力有效。

成绩来之不易，这是习近平新时代中国特色社会主义思想生动实践的结果，是党中央、国务院和省委、省政府、市委坚强领导的结果，是全市人民团结奋斗和各方面大力支持的结果。在此，我代表漳州市人民政府，向全市人民，向人大代表、政协委员、各民主党派、工商联、各人民团体、离退休老同志和各界人士，向驻漳省部属单位、驻漳部队、武警官兵，企业界人士和来漳创业务工人员，向关心支持漳州发展的港澳台同胞、海外乡亲和国际友人，表示衷心的感谢！

前进的道路从不会一帆风顺。我们也清醒认识到发展中的困难和工作中存在的问题，主要是：经济下行压力有所加大，部分企业生产经营困难增多，新动能对高质量发展支撑不够强劲；生态环境保护压力较大;教育、医疗补短板力度推进不大，还不能满足社会需求；政府职能转变不到位，营商环境离市场主体期待还有差距。面对新时代、新要求，我们必须直面差距、敢于担当，采取切实有效的措施加以解决。

二、坚持高质量发展落实赶超，扎实做好2019年工作

今年是新中国成立70周年，是决胜全面建成小康社会、坚持高质量发展落实赶超的关键一年。我们要以习近平新时代中国特色社会主义思想为指导，全面贯彻党的十九大和十九届二中、三中全会精神，认真贯彻落实中央经济工作会议、省委十届六次七次全会、市委十一届七次八次全会和省委、市委经济工作会议的决策部署，紧紧围绕统筹推进“五位一体”总体布局和协调推进“四个全面”战略布局，坚持稳中求进工作总基调，坚持新发展理念，坚持高质量发展落实赶超，坚持以供给侧结构性改革为主线，坚持深化市场化改革、扩大高水平开放，统筹推进稳增长、促改革、调结构、惠民生、防风险工作，进一步稳就业、稳金融、稳外贸、稳外资、稳投资、稳预期，持续深化“三抓三比、十项竞赛”，抓牢抓实增长点，奋力赶超作贡献，一二三产齐发力，重中之重抓古雷，继续打好三大攻坚战，持续延展“生态+”效益，加快建设富美新漳州，奋力当好新福建建设先锋，以优异成绩庆祝新中国成立70周年。

2019年，全市经济社会发展主要预期目标是：地区生产总值增长9%左右；一般公共预算总收入增长7%左右，地方一般公共预算收入增长4%左右；固定资产投资增长12%左右；规模工业增加值增长9.8%左右，规模工业总产值增长10.1%左右；外贸出口增长3%左右；实际利用外资增长3.5%左右；社会消费品零售总额增长11%左右，居民消费价格总水平涨幅控制在3%左右；城镇登记失业率控制在3.6%以内；城镇和农村居民人均可支配收入分别增长8.6%、9%左右；落实节能减排降碳任务。

（一）扩大有效供给，促进产业高质量发展

加快制造业转型升级。围绕中央提出的“巩固、增强、提升、畅通”八字方针，深化供给侧结构性改革，促进实体经济创新转型。实施重点产业建链强链补链工程，支持组建产业联盟，发展壮大“4+4”产业集群。围绕石化、新材料、新能源等产业，精准制定扶持政策，挖掘培育一批有基础、有特色、有成长性、有贡献率的优势产业。实施产业龙头改造升级计划和新一轮企业技改专项行动，力争新增（新上）规模工业企业100家，技改投资增长15%。尽快开工建设食品总部产业园区、时间小镇，扩大“中国食品名城”“中国钟表名城”品牌效益。进一步修订完善支持建筑业发展政策，促进建筑业实现更大发展。

大力培育新兴产业。推进新兴产业倍增工程，设立新兴产业股权投资基金，加快发展新一代信息技术、生物医药、节能环保、海洋高新、医养健康等产业。加快高新区物联网产业园、立达信科技小镇等项目建设，大力发展物联网、大数据、虚拟现实等数字经济。在制造、金融、医疗、安防、政务等领域，建设一批人工智能应用示范项目。加大引办军民融合产业项目，建设军民融合产业园区。支持建设海峡两岸（漳州）设计创意中心，潜心培育工业设计，吸引优秀设计团队来漳创业，逐步打响漳州制造、漳州创造品牌。

壮大提升现代服务业。实施现代服务业提速提质工程，推动服务业扩规模提质量。现代物流重点推进台商区保税物流中心（B型）、中国智能骨干网、漳龙物流园等项目建设，提升运输、仓储、配送、电商平台、供应链金融等全要素服务功能，推动物流业资源整合、块状布局、降本增效。金融服务重点加快推进金融集中区建设，发展绿色金融、普惠金融，深化“引银入漳”，完善政银企对接长效机制，加大民营企业、小微企业信贷扶持力度。全域旅游重点提升旅游六大要素，优化精品旅游线路，开发“月光经济”产品，强化精准营销，持续打响花样漳州品牌。推动文旅深度融合，大力发展文化产业，培育动漫、创意设计、文物博物等新型文化业态。

旗帜鲜明支持民营经济发展。坚持“两个毫不动摇”“三个没有变”，牢记“民营企业和民营企业家是我们自己人”，强化一企一策精准帮扶，真心实意为民营企业排忧解难，营造懂民企、爱民企、帮民企的良好氛围。深化“政策不睡觉、落地见成效”活动，不折不扣落实民营经济扶持政策。专项治理涉企收费和拖欠民企资金问题，落实更大规模减税和更明显的降费。

营造良好创新环境。加快实施创新能力突破行动，推动产业、创新、人才、资金、政策相互融合，力争全市研发投入增长22%以上。鼓励企业建设国家和省级重点实验室、工程技术研究中心，新增创新研发平台2家以上、高新技术企业和创新型企业25家、科技小巨人20家。支持古雷开发区与厦门大学共建化工研发中心和中试基地。对标科创板要求，创造条件支持高新技术企业上市。实施招才引才个性化政策，坚持院士工匠一起引、培养使用两手抓，进一步扩大人才“朋友圈”。

（二）强化项目带动，夯实赶超发展后劲支撑

深化“五个一批”项目攻坚。完善市县领导抓重特大项目机制，强化“一月一协调、一季一调度、一季一次集中开工”，充分发挥“一把手”既当指挥员、又当“施工队长”的作用，促进项目建设提速提效。实施“十百千支撑工程”，更加突出工业投资，今年安排重点项目596个、年度投资1468亿元，其中工业项目225个，推动漳州核电、奇美化工、三宝钢铁产能置换改造等200个项目落地开工，加快推进中海油漳州LNG、福欣节镍不锈钢等项目建设，力促猛狮锂离子电池等150个项目建成或部分建成。继续实施一批投资工程包和十大惠民特色工程包。运用市场化手段和要素价格杠杆，大力破解项目用地、用林、用海、用电及节能减排指标等制约因素，提升项目立项、落地时效。

提升招商引资实效。着力延长产业链、做强产业群，加强项目策划力量，组建专业化项目策划团队，谋划生成一批重点储备项目。瞄准世界500强、行业龙头、大型民企，加大小分队招商、精准招商，力争重大项目招引实现新突破，新签约项目250个以上、总投资1000亿元以上。发挥亲情乡谊优势，重视商会招商、台侨招商，促进更多漳籍企业家返乡投资。围绕2020年出产值、出效益的目标，紧盯资金到位率、项目开工率和竣工投产率三大关键指标，推动签约项目加快落地建设。

加大基础设施投入。抓住国家加大基础设施投入机遇，加快策划一批重大基础设施项目，增强吸纳要素、承载项目的能力。聚焦打通关键通道和重要节点，开工建设“九龙云巴”、厦门地铁6号线漳州段，加快建设福厦（漳）高铁、漳武高速、24个城市道路“互联互通”项目以及古雷航道三期、后石航道二期等项目，推进漳汕高铁、沈海高速改扩建工程、厦漳泉城际轻轨R1线等前期工作。推进古雷石化基地和台商投资区等园区热电联产、集中供热。实施一批涉农投资工程包，深化“四好”农村公路建设，加大水利设施建设，推动农村基础设施提档升级。实施新一轮农网升级改造，加快输变电工程建设，完善智能配电网。

（三）突出重点区域，提高协同发展质量水平

打造古雷增长新引擎。围绕“三稳三突破”推进古雷高质量发展，加速释放增长效益，向着世界一流石化基地迈进。做好石化产业筑链建群文章，推动PX稳定生产、PTA和春达化工尽快达产、海顺德特种油品复产，推进古雷炼化一体化一期、中怡精细化工一期等重大项目加快建设，古雷炼化一体化二期、省石化与沙特合作项目等早日落地。更加重视招商选资、招商选税，洽谈引进一批中下游石化项目，实现石化全产业链发展。加快完善码头泊位、公共管廊、污水厂、供电设施等公用工程，推进精细化工园区建设，提升服务配套水平。

拓展湾区发展新空间。主动融入闽西南协同发展区，加快串联厦门湾、东山湾两大湾区，推进一批重大协作项目建设，加速基础对接、产业协同、融合发展。强化厦漳同城化先行示范区引领带动，规划建设同城化产业园，吸引更多人流、物流、资金流、信息流往湾区聚集，打造环湾经济发展带。统筹抓好沿海大通道两侧区域的规划提升、基础投入、土地收储、产业布局，激活漳州南部片区发展活力。注重项目合作、资源导入、体制创新，深化与招商局集团新一轮战略合作，共建高端产业集聚区。加快发展临港工业，培育壮大港口经济。

增强县域经济新活力。强化市域统筹，推进市县经济发展、城乡建设、土地利用、生态保护等规划“多规合一”，加快构建产业互补、平台共享、设施共建、互联互通的融合发展格局。支持各地立足资源禀赋，坚持绿色发展、错位发展，把优势产业做出特色、做出规模，每个县（市、区）集中突破若干个重点优势产业。围绕扩权强县，持续加大政策扶持、人才支持、要素保障力度。创新开发区管理体制和运行机制，完善考核评价和激励约束，推动开发区高质量发展。

（四）坚持“生态+”引领，建设宜居宜业美丽家园

做活城市经济。做好“生态+”的经济文章，把产业加进去、效益做出来，把文化加进去、旅游带起来，把服务加进去、品质提上来，让绿水青山成为金山银山。围绕提高中心城区繁荣度，丰富城市业态，打造“五湖四海”新商圈。科学布局文创产业，开发利用旧厂房、旧车间，打造一批创意产业街区、文化艺术街区、餐饮娱乐街区。加大闲置土地存量清理、挖潜增效，推动工业用地转型升级、退二进三，推进城郊开发区创新转型。盘活闲置商业办公用房资源，培育发展楼宇经济、总部经济。坚持稳地价、稳房价、稳预期，完善土地、住房供应保障体系，促进房地产市场平稳健康发展。统筹市区及周边场馆、景点、酒店等资源，扶持发展会展经济和会奖旅游。优化城市商业布局，做强做特专业市场。

做精城市品质。运用“多规合一”成果，启动“城市双修”专项规划及重要节点、片区、地段城市设计，加快建设西湖生态园、丹霞驿站、南湖温泉酒店等项目，有效发挥“生态+”效益，加速“一江两岸”聚人气、共繁荣。以生态价值规划高新区成长坐标，加快推进一批高新技术、现代服务业项目落地。实施“五湖四海”绿道连接工程，打通串联城市的慢行系统。注重把文化融入城市设计，打响历史名人文化品牌，加快古城修缮提升、业态植入，增强历史文化名城活力魅力。实施老旧小区改造工程包，加大占用河道、道路等公共空间治理，推进一批背街小巷“微改造”，加快城市有机更新。深化全国文明城市创建，开展“中心城区城市管理提升年”活动，推进智慧城管建设，提升城市管理精细化水平。

做优城市功能。开展市区交通畅通工程，加快中心城区快速交通系统、环城路网建设，实施一批道路提升改造，打通3条断头路，新增3000个停车泊位，努力解决停车难、出行堵问题。实施农贸市场改造建设三年行动，逐步推动农贸市场环境根本好转。深化黑臭水体治理示范城市建设，推进清淤疏浚、截污处理、源头活水，加快实现河畅水清、岸绿景美。完善提升排水防洪设施，建设海绵城市。实施提升城市供水水质三年行动，加大饮用水源地保护，加快第二、第三水厂建设，推进城乡供水一体化，确保群众饮水安全。

（五）全面实施乡村振兴战略，扎实推进农业农村现代化

抓特色农业，促产业振兴。坚持品种引领、品质提升、品牌打造，实施特色现代农业“六百工程”，做大做强九大优势特色产业。扶持壮大“三朵花、三泡茶、四珍菌、六条鱼、十大果”，新增、续展“三品一标”50个。加大水仙花原产地保护和产业发展，擦亮打响漳州水仙花品牌。做大农产品精深加工业，培育扶持原料基地建设，促进一二三产融合发展。加快水稻生产功能区建设，巩固提升粮食产能，确保粮食安全。提升农业“两区”建设水平，实施农业绿色发展行动，整市推进畜禽养殖废弃物资源化利用，打造农业绿色发展漳州样板。加强重大动植物疫情防控，确保农业生产安全。

抓脱贫攻坚，促巩固提升。开展打赢脱贫攻坚战稳定提质行动，保持政策不变、力度不减、队伍不撤，聚焦新增和已脱贫但基础较脆弱的贫困人口，重点解决好实现“两不愁、三保障”面临的突出问题，促进稳定脱贫、防止返贫。全面落实强农惠农政策，支持农民就业创业，提高农民收入水平。完善科技特派员制度，加快培育新型职业农民和农村实用人才。持续推进“养鸡生蛋”工程、“三千”工程，落实新增建档立卡贫困人口进退机制，探索缓解相对贫困人口扶贫机制，确保断“穷根”、奔小康。加强扶贫领域联合监督检查，加大扶贫资金在线监管，以作风攻坚促脱贫攻坚。

抓规划引领，促乡村宜居。坚持乡村振兴规划先行，注重留白、留绿、留旧、留文、留魂，深化“345”示范工程，加快建设美丽宜居乡村。打好农村人居环境整治“一革命四行动”，深化铁路沿线环境综合整治、“百路千村”两违综合治理，梯次推进乡村山水林田路房整改提升。加强农村住房规划管理，加大农村危房改造力度。落实农村土地“三权”分置，深化农村集体产权、林权制度改革。坚持人才振兴，培育挖掘新乡贤。健全农村基层服务体系，推进移风易俗，促进乡风文明。

（六）着力先行先试，推动新时代改革开放再出发

拓展改革广度深度。认真落实省委、省政府《关于进一步深化改革扩大开放的若干措施》，对标一流，奋力先行，在更高起点上推动新时代改革开放再出发。全面完成机构改革任务，确保实现流畅运转、高效融合。更高标准推进营商环境建设，深化“放管服”改革，全面清理证明事项，推动“漳州通”APP上线投用，促进环境提优、效能提升、服务提质。加强国资监管，提高国有资本效率，推进国有企业治理现代化。全面实施预算绩效管理，加大财政资金监督检查，加快政府采购制度改革。深化统计制度改革，扎实开展第四次全国经济普查工作。

提升开放层次水平。主动融入海丝核心区建设，创新推进“漳州味·世界行”，支持企业开展国际化经营。积极应对国际经贸形势变化，引导外贸企业转型升级，提升出口质量效益。发展壮大市场采购、跨境电商等外贸新模式，扩大先进技术装备、紧缺资源和优质消费品进口，推动买全球、卖全球。全面实施一站式作业、一体化通关管理，提升贸易便利化水平。优化口岸功能布局，完善港航基础设施，争取更多货源从漳州港口进出。加强与港澳经贸合作，涵养新生代华人华侨资源。

深化对台交流合作。深入学习宣传习近平总书记在《告台湾同胞书》发表40周年纪念会上的重要讲话精神，围绕做深做实漳台融合文章，深化“四个一”工程，推进“同城待遇”应享尽享，提升台胞归属感和认同感。深化漳台产业对接，拓展技术、管理、项目、服务等交流合作。加快推进台资企业资本项目管理便利化试点，支持台资企业增资扩产深扎根。提升对台展会实效，密切漳台人文交流，吸引更多台商台青来漳创业就业。完善市台胞台商服务中心功能，推广设立台胞服务窗口，维护台胞台商合法权益。

（七）加快民生补短板，努力提高群众生活质量

打好污染防治攻坚战。以打翻身仗的决心和毅力深化“三铁治污、提升三质”，打好污染防治八大标志性战役，持续抓好中央、省环保督察反馈问题整改，确保主要污染物排放总量明显减少。坚决打好蓝天保卫战，注重源头管控、联防联控，狠抓扬尘和臭氧治理，全面整治“散乱污”企业，实现空气质量根本好转。打好碧水保卫战，全面落实河湖长制，攻坚整治污染源，确保流域水质持续改善。打好净土保卫战，加大土壤污染治理修复，强化工业固废综合利用，推进垃圾分类处置，建成投用2座生活垃圾焚烧发电厂。

打好防范化解重大风险攻坚战。重点是防范化解金融风险，坚持结构性去杠杆基本思路，落实属地责任，一企一策、一链一策精准化解，严厉打击恶意逃废债、非法集资等违法犯罪行为，确保全市不良贷款率控制在2%以内，守住不发生系统性金融风险底线。按照“坚定、可控、有序、适度”的要求，规范政府举债行为，强化债务风险防控，降低市属国企平均资产负债率。

优化公共服务供给。扩大优质教育资源覆盖面，新建公办幼儿园40所，开工建设市实小龙文校区，新增幼儿园学位10800个；深化高职院校产教融合、校企合作，加大产业急需专业扶持力度；加强新时代教师队伍建设，落实立德树人根本任务，提高教育教学质量；规范民办教育发展。推进优质医疗资源扩容，深化医药卫生体制改革，加快建设市医院新总院，支持县级医院发展，提升基层卫生服务能力，新增医疗床位1200张以上；大力引进医疗卫生人才，健全妇幼健康服务体系，传承发展中医药事业；支持社会资本办医，推进泰禾医院、天福医院等项目建设。盘活乡村敬老院资源，全市新增养老床位1000张以上。支持文化繁荣发展，加快基层综合文化中心、县级综合档案馆建设，打造更多文艺精品和群众文化品牌。做好《漳州市志（1991-2007）》出版工作。开展全民健身活动，整合体育设施资源，加速体育产业市场化运营。

完善社会保障体系。实施32项为民办实事项目。做好高校毕业生、失业人员、失地农民等就业帮扶，新增城镇就业4.6万人。完善低保标准自然增长机制，落实大病、失业、工伤等保险制度。推进西湖生态园片区、长福片区等棚户区改造，新建保障性安居工程15130套。完善城乡社会救助体系，加强城乡困境儿童保障，发展残疾人事业、社会福利和慈善事业，保障妇女儿童、青少年、老年人合法权益。

维护社会安定稳定。纵深推进扫黑除恶专项斗争，开展突出治安问题专项整治，健全防范化解风险隐患责任链条，提升“平安漳州”建设水平。压紧压实安全生产责任，推进重点行业领域专项整治，加快构建安全风险分级管控和隐患排查治理双重预防机制，坚决遏制较大及以上生产安全事故发生。创新食品药品监管方式，用“四个最严”确保人民群众舌尖上的安全，打造中国安全食品名城。完善应急管理体制机制，做好防汛防台、消防、地震、气象、地质等工作，提高政府应对突发事件能力和防灾减灾救灾能力。

同时，要加强国防教育、国防动员、人防、海防和双拥共建，做好退役军人优抚安置。全面贯彻党的宗教工作基本方针，帮扶少数民族乡村加快发展。支持工会、共青团、妇联、残联、科协、红十字会等人民团体发挥更大作用。

三、加强政府自身建设，提升推动高质量发展落实赶超的能力

全面推进政府政治、思想、作风、纪律建设，打造忠诚干净担当的高素质专业化公务员队伍。

坚守讲政治的生命线。坚持以党的政治建设为统领，牢固树立“四个意识”，坚定“四个自信”，坚决做到“两个维护”，把讲政治的要求贯穿到政府工作的全过程。持续深化“大学习”活动，推动习近平新时代中国特色社会主义思想入脑入心、走深走实。坚定不移贯彻落实党中央、国务院和省委、省政府、市委各项决策部署，以实际行动践行对党的绝对忠诚。

提振当先锋的精气神。大胆解放思想，勇于开拓创新，以敢为人先的责任担当、敢于亮剑的斗争精神，坚定当先锋、促赶超的信心决心。加强学习实践，提升“八大本领”，增强干部创造力和执行力。带着深厚感情为民谋利造福，精准查补民生短板，让群众有更多获得感幸福感。落实正向激励、容错纠错机制，关心关爱基层干部，激发广大干部新时代新担当新作为。

强化守法律的硬约束。弘扬宪法精神，推进依法行政，加大全民普法力度。做好法规草案起草工作，适时向市人大常委会提出议案。健全依法决策机制，加强规范性文件备案审查和重大行政决策合法性审查。加强行政执法监督，提升行政复议、行政应诉能力。落实“政府承诺+社会监督+失信问责”机制，建设诚信政府。自觉接受人大及其常委会监督、政协民主监督、监察机关监督、社会和舆论监督，提高建议提案办理实效。

打造高效率的软环境。践行“马上就办、真抓实干”，坚持重实干、见实效，坚持说了算、定了干，形成狠抓落实、善做善成的鲜明导向。对标最高标准，聚焦企业关切，加快打造一流营商环境，让“办事讲程序、高效率、不求人”成为习惯、成为风气。真心实意帮扶企业，禁止对企业一切不必要的检查、督查和考核，给企业留足“宁静时间”生产经营。

筑牢不敢腐的防洪堤。坚持把纪律和规矩挺在前面，认真贯彻“八个坚定不移”要求，全面落实从严治党主体责任。严格执行中央八项规定及其实施细则和省市实施办法，紧盯不敬畏、不在乎、喊口号、装样子的问题，坚决破除形式主义、官僚主义。深入推进反腐败斗争，支持纪检监察机关履行职责，推动审计监督全覆盖，保证干部清正、政府清廉、政治清明。

各位代表，当前，漳州正处在坚持高质量发展落实赶超的关键时期，使命光荣、责任重大！让我们高举习近平新时代中国特色社会主义思想伟大旗帜，在省委、省政府和市委的坚强领导下，锐意进取、扎实苦干，为加快富美新漳州建设、当好新福建建设先锋而努力奋斗!
